# Heat Stress Weakens the Skin Barrier Function in Sturgeon by Decreasing Mucus Secretion and Disrupting the Mucosal Microbiota

**DOI:** 10.3389/fmicb.2022.860079

**Published:** 2022-04-26

**Authors:** Shiyong Yang, Wenqiang Xu, Chaolun Tan, Minghao Li, Datian Li, Chaoyang Zhang, Langkun Feng, Qianyu Chen, Jun Jiang, Yunkun Li, Zongjun Du, Wei Luo, Caiyi Li, Quan Gong, Xiaoli Huang, Xiaogang Du, Jun Du, Guangxun Liu, Jiayun Wu

**Affiliations:** ^1^Department of Aquaculture, College of Animal Science and Technology, Sichuan Agricultural University, Chengdu, China; ^2^College of Life Science, Sichuan Agricultural University, Ya’an, China; ^3^Fisheries Institute, Sichuan Academy of Agricultural Sciences, Chengdu, China

**Keywords:** heat stress, Siberian sturgeon, skin, mucous cells, microbial diversity

## Abstract

Heat stress induced by global warming has damaged the well-being of aquatic animals. The skin tissue plays a crucial role as a defense barrier to protect organism, however, little is known about the effect of heat stress on fish skin, particularly in cold-water fish species. Here, we investigated the effects of mild heat stress (24°C, MS) and high heat stress (28°C, HS) on Siberian sturgeon skin using RNA-seq, histological observation, and microbial diversity analysis. In RNA-seq, 8,819 differentially expressed genes (DEGs) in MS vs. C group and 12,814 DEGs in HS vs. C group were acquired, of which the MS vs. C and HS vs. C groups shared 3,903 DEGs, but only 1,652 DEGs were successfully annotated. The shared DEGs were significantly enriched in pathways associating with mucins synthesis. Histological observation showed that the heat stresses significantly reduced the number of skin mucous cells and induced the damages of epidermis. The microbial diversity analysis elicited that heat stress markedly disrupted the diversity and abundance of skin microbiota by increasing of potential pathogens (*Vibrionimonas*, *Mesorhizobium*, and *Phyllobacterium*) and decreasing of probiotics (*Bradyrhizobium* and *Methylovirgula*). In conclusion, this study reveals that heat stress causes adverse effects on sturgeon skin, reflecting in decreasing the mucus secretion and disordering the mucosal microbiota, which may contribute to develop the preventive strategy for heat stress caused by global warming.

## Introduction

In the past few decades, the increasingly pronounced global warming has gradually warmed the Earth’s water temperature (Rossati, 2017; Alfonso et al., 2021). The increasing water temperature is devastating for fish species, particularly in cold-water fish species that are poikilothermic vertebrates and particularly vulnerable to the change of environmental temperature. Excessively high or low water temperature can cause physiological dysfunction and even death in fishes (Little et al., 2020). In this case, the heat stress caused by global warming restricts the development of aquaculture primarily by impacting fisheries ecosystem and economic benefit (Brander, 2007; Ng’onga et al., 2019).

In aquatic environment, fish health is susceptible to the change of environmental factors like water temperature. To cope the possible threats in water environment, fishes have generated an important immune barrier skin (Glover et al., 2013; Gomez et al., 2013). Skin tissue, including its surface, is the first defender of contacting fish body to water environment, which contributes to combat against the adverse effects, such as stresses and pathogens (Llewellyn et al., 2014). Skin mucus is a strong sticky substance secreted by epidermal mucous cells, which covers the surface of fish skin, forming an effective protective barrier (Fæste et al., 2020). And, skin mucous is colonized by a complex community of microbiota that plays an essential role to resist environmental pathogens (Ellis, 2001; Minniti et al., 2017). Fish skin mucosal microbiota gives the contact of fish with surrounding water, but variations in water physico-chemistry drives microbial dysbiosis (Sylvain et al., 2016). Mohammed and Arias (2015) reported that potassium permanganate caused changes in the skin microbiota of catfish and raises susceptibility to columnar disease, and Sylvain et al. (2016) study found that a drop in pH caused changes in the skin microbiota of Amazonian fish, suggesting that skin mucosal microbiota can be used as a marker to monitor fish health and improve their growth performance through rapid diagnosis. Unfortunately, the heat stress-derived variations in mucus secretion and mucosal microbiota in fish skin are poorly understood.

Siberian sturgeon (*Acipenser baerii*) is widely farmed around the world because of richness of its flesh in unsaturated fatty acids and protein and high quality caviar (Bronzi and Rosenthal, 2014; Bronzi et al., 2019). The optimal living temperature for Siberian sturgeon is around 20°C (Secor and Gunderson, 1998; Kappenman et al., 2009; Silvestre et al., 2010). Warm water (24°C), or excessive temperature water (28°C), was proved to derive stress response in Siberian sturgeon, eventually leading to the collapse of immunity and even death (Castellano et al., 2017; Yang et al., 2021). However, whether and how heat stress poses a threat to the skin of cold-water fish, such as sturgeon, remains to be explored. In this study, we firstly performed RNA-Seq to uncover the gene expression features in skin tissue of Siberian sturgeon suffered from heat stresses at 24 and 28°C. The histological observation was carried out to assess the pathological change in the skin of Siberian sturgeon under the heat stresses. Additionally, we analyzed the community structure of the skin microbiota by high throughput sequencing. This study aims to reveal the preliminary mechanism of the effect of heat stress on fish skin.

## Materials and Methods

### Experimental Fishes

A total of 90 healthy juvenile Siberian sturgeon (143.6 ± 4.6 g) were supplied from a sturgeon aquafarms (Hongya Donglong Sturgeon Industry Co., Ltd., Leshan, China). The fishes were then bred in circular glass tanks (1.5 m diameter and 1 m height) in laboratory. During fish domestication, water quality parameters including temperature, dissolved oxygen and pH were maintained at 20 ± 1.0°C, 7.1 ± 0.8 mg⋅L^–1^ and 7.8 ± 0.3, respectively, and fishes were fed with commercial diet (3% of fish weight) three times per day (at 8:00 am, 14:00 pm, and 20:00 pm, respectively). All animal handling procedures were approved by the Animal Care and Use Committee of Sichuan Agricultural University, following the guidelines of animal experiments of Sichuan Agricultural University under permit number AW-2020302179.

### Heat Stress Treatment

The optimum growth status for Siberian sturgeon has been reported to be about 20°C (Aidos et al., 2020), but the living temperature raising to 24 and 28°C caused distinct stress response and thus effected the survival rate during sturgeon farming (Castellano et al., 2017; Aidos et al., 2020; Yang et al., 2021). After 2 weeks acclimatization under laboratory condition, 90 fishes were randomly divided into three groups, each group includes three tanks, 10 fishes per tank. The first group was the control group (C), the water temperature was kept at 20.0 ± 1.0°C. Fishes in the second and third groups underwent mimetic long-term heat stresses, mild heat stress (MS) and high heat stress (HS), by raising slowly from 20°C ± 1.0°C to 24°C ± 1.0°C and 28°C ± 1.0°C in 1 week, respectively, and kept the temperatures for 1 week.

### Sample Collection

After the heat stress processes, the skin tissues of all fishes were aseptically collected. Briefly, six fishes from each group were randomly anesthetized by MS-222. The skin tissues were aseptically excised from both the dorsal side and ventral side and stored at −80°C until for use. Of these six skin samples, three were used for RNA-seq, while the rest were used for microbial diversity analysis. To preserve the integrity of skin epithelial cells, the skin samples jointed with subcutaneous muscle from random three fishes were collected and fixed in 10% neutral buffered formalin for at least 24 h, until for histological observation.

### Histopathological Observation

The fixed skin tissues were dehydrated through a conventional alcohol gradient, then cleared in xylene and embedded in paraffin wax. Sections of 5 μm were prepared and mounted on slide for hematoxylin and eosin (H&E) and periodic acid Schiff (PAS) staining. After neutral sealing with glue, the slides were observed under an optical microscope (Nikon, Tokyo, Japan). The number of mucous cells was measured in 10 visual fields and then calculated the average.

To evaluate the effects of heat stress on the skin of Siberian sturgeon, we evaluated the skin damage based on a scoring system proposed by Baums et al. (2013). Briefly, abnormal changes in the epithelial layer of the skin, such as hyperplasia, necrosis, deposits, hypertrophy, hyperemia, or atrophy, were assessed with using a score of 0–5: (0) unchanged; (1) mildly altered; (3) moderately altered; and (5) severely altered (diffuse necrosis).

### RNA-Seq

#### RNA Extraction, Library Construction, and Sequencing

To explore the gene expression features of skin under the heat stresses, RNA-seq was performed. According to the manufacturer’s instructions (Invitrogen, Carlsbad, CA, United States), TRIzol^®^reagent was used to extract total RNA from the skin samples, and DNase I (Takara, Dalian, China) was used to remove genomic DNA. Then, the RNA quality and quantification were determined by 2100 Bioanalyzer (Agilent Technologies, Santa Clara, CA, United States) and ND-2000 (NanoDrop Technologies, Shanghai, China), respectively. High-quality RNA samples were selected to construct sequencing libraries by TruSeq™ RNA sample preparation Kit (San Diego, CA, United States). The libraries were sequenced on an Illumina NovaSeq 6000 sequencing platform (Shanghai Biozeron Biotechnology Co., Ltd).

#### *De novo* Assembly and Annotation

The raw paired end reads were firstly trimmed and controlled the quality by Trim Galore and Fast QC software to acquire clean data. Then, the clean data from all samples were used to *de novo* assembly by Trinity^1^. All the assembled transcripts were searched against the NCBI protein non-redundant (NR), String, and KEGG databases using BLASTX to identify the proteins that had the highest sequence similarity with the given transcripts to retrieve their function annotations and a typical cut-off *E*-values less than 1.0 × 10^––5^ was set up. Blast2GO program^2^ was used to get Gene Ontology (GO) annotations of unique assembled transcripts for describing biological processes, molecular functions, and cellular components. Metabolic pathway analysis was performed using the Kyoto Encyclopedia of Genes and Genomes (KEGG)^3^.

#### Differential Expression and Functional Enrichment Analyses

Reads per kilobase of exon per million mapped reads (RPKM) value was used for identification of differentially expressed genes (DEGs) amongst the treatments. RSEM software was used to quantify gene and isoform abundances. Differential expression analysis was performed using the DEGSeq R statistical package software. In addition, GO functional enrichment analysis of DEGs and KEGG pathway analysis were implemented by Goatools^4^ and KOBAS^5^.

### Microbial Diversity Analysis

#### DNA Extraction and Purification

Microbial DNA was extracted from Siberian sturgeon skin using the E.Z.N.A.^®^DNA Kit (Omega Bio-Tek, Norcross, GA, United States), according to manufacturer’s protocols. After extraction, the V1-V9 region of samples were amplified by polymerase chain reaction (PCR) with the forward primers (27F: 5′-AGRGTTYGATYMTGGCTCAG-3′) and the reverse primer (1492R: 5′-RGYTACCTTGTTACGACTT-3′). Then PCR product was extracted from 2% agarose gel and purified with the AxyPrep DNA Gel Extraction Kit (Axygen Biosciences, Union City, CA, United States) according to the manufacturer’s instructions.

#### Sequencing and Processing

The libraries were prepared from the amplified DNA by blunt-ligation according to the manufacturer’s instructions (Pacific Biosciences). Purified libraries from the Zymo and HMP mock communities were sequenced on dedicated PacBio Sequel II 8M cells using the Sequencing Kit 2.0 chemistry. All amplicon sequencing was performed by Shanghai Biozeron Biotechnology Co., Ltd. (Shanghai, China). PacBio raw reads were processed using the SMRT Link Analysis software (version 9.0), the dataset was prepared for analysis by excluding that: (i) shorter than 50 bp, (ii) <10 bp in libraries, (iii) ambiguous nucleotides that constituted over 20% of the sequence. The remaining sequences were clustered into operational taxonomic units (OTUs) with 98.65% similarity cutoff using UPARSE (version 7.1)^6^ and chimeric sequences were identified and removed using UCHIME. The phylogenetic affiliation of each 16S rRNA gene sequence was analyzed by RDP Classifier^7^ against the SILVA (SSU132) 16S rRNA database using confidence threshold of 70% (Amato et al., 2013).

### Data Analysis

All data are expressed as mean ± standard deviation. Significances between groups were analyzed by one-way analysis and *t*-test. Statistical analyses were performed using SPSS 22.0 software (IBM Corp., Armonk, NY, United States). *P*-value < 0.05 was considered significant for all tests.

## Results

### Characteristics of RNA-Seq Data and Assembly Annotation

Nine RNA-seq libraries were constructed from the skin of Siberian sturgeon under the heat stresses, and three biological replicates were set up for each group. After filtering, the number of clean reads in each library ranged from 24 to 34 million, and the clean Q30 values ranged from 93.17 to 93.85% per sample, indicating that the sequencing data were of high quality and of significance for further analysis (Table 1). All datasets from the Illumina sequencing platform can be found in the National Center for Biotechnology Information Short Read Archive Database under Accession number (PRJNA731649).

**TABLE 1:** The sequence quality and mapping results in the nine samples.

Library	Raw reads	Clean reads	Clean reads rate (%)	Mapped reads	Mapping rate (%)	%≥Q30
C_1	38822614	28325680	72.96	20743801	73.23	93.85
C_2	40084474	31885398	79.55	22613666	70.92	93.72
C_3	38169848	29024536	76.04	21099270	72.69	93.71
MS_1	37562062	27971210	74.47	20599705	73.65	93.17
MS_2	37444736	24392574	65.14	18009939	73.83	93.79
MS_3	42465586	30839452	72.62	22401870	72.64	93.22
HS_1	37960000	28274794	74.49	20982554	74.21	93.63
HS_2	43243716	34618004	80.05	25496956	73.65	93.55
HS_3	39392218	29463872	74.80	21416168	72.69	93.74

To fully understand the gene functional information, the unigenes were annotated in five databases (Supplementary Figure 1B). Finally, 172,158 unigenes with annotation information were obtained. The GO annotation statistic showed that most unigenes were annotated to biological processes and cellular components (Supplementary Figure 1C). The KEGG pathway analysis revealed that the majority of the unigenes were annotated to signal transduction and endocrine system (Supplementary Figure 1D).

### Identification of Differentially Expressed Genes and Gene Ontology Enrichment Analysis

A total of 28,072 differentially expressed genes (DEGs) were detected in the three comparison groups (MS vs. C, HS vs. C and HS vs. MS). 4,182 upregulated DEGs and 4,636 downregulated DEGs were detected in the MS vs. C group, while 4,673 upregulated DEGs and 8,140 downregulated DEGs were detected in the HS vs. C group. In addition, 2,692 upregulated DEGs and 3,749 downregulated DEGs were detected in the HS vs. MS group (Supplementary Figure 2). To elaborate the effects of the heat stresses on Siberian sturgeon skin, we selected the top 30 significantly enriched terms of the GO enrichment for analysis. A large number of DEGs in the MS vs. C group were categorized to “responses to cAMP,” “keratinocyte differentiation,” “keratinization,” “responses to unfolded proteins,” and “defense responses to bacteria.” The “regulation of chemokine production,” “responses to unfolded protein,” “keratinocyte differentiation,” “cellular responses to a mechanical stimulus,” and “skin development categories” dominated the HS vs. C group. In HS vs. MS group, the categories “regulation of sequestering of calcium ion” and “response to calcium ion” were highly enriched (Supplementary Figure 3).

### Kyoto Encyclopedia of Genes and Genomes Pathway Analysis

**FIGURE 1:**
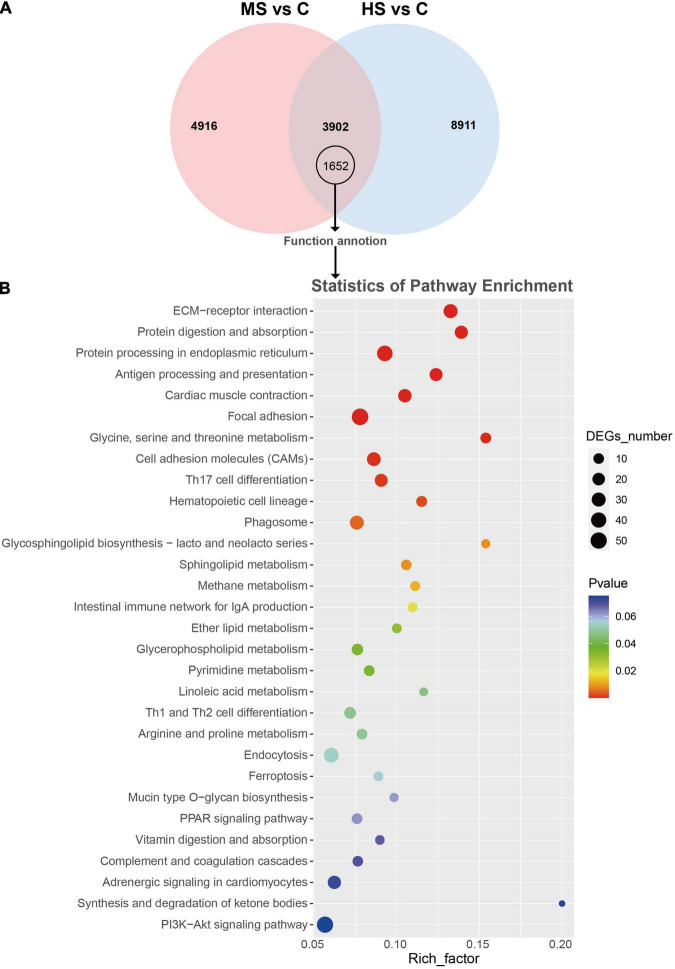
Venn diagram, KEGG enrichment analysis of the shared DEGs in MS vs. C and HS vs. C groups. **(A)** Venn diagram showed the 8,818 in MS vs. C group and 12,813 DEGs in HS vs. C group, and 1,652 shared annotated DEGs. **(B)** Scatter diagram of KEGG enrichment analysis of the shared DEGs. *X*-axis (enrichment factor) indicates the enrichment level of DEGs in each pathway, while *Y*-axis represents the reliability of enrichment analysis of each pathway.

**FIGURE 2:**
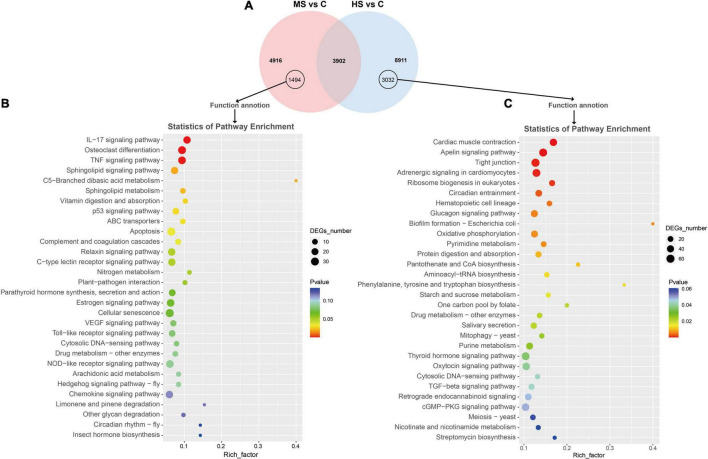
Venn diagram and KEGG enrichment analysis of the non-shared DEGs in MS vs. C and HS vs. C groups. **(A)** Venn diagram showed 1,494 and 3,032 non-shared annotated DEGs in MS vs. C and HS vs. C groups, respectively. **(B,C)** Scatter diagram of KEGG enrichment analysis. *X*-axis (enrichment factor) indicates the enrichment level of DEGs in each pathway, while *Y*-axis represents the reliability of enrichment analysis of each pathway.

In order to analyze the genes that responded to different temperatures, the Venn diagrams were drawn (Figure 1A). Two comparing groups shared 3,903 DEGs that included 1,652 function annotated genes. The KEGG enrichment of these annotated genes was then visualized by bubble chart according to their FPKM values (Figure 1B). Notably, the genes related to protein synthesis (e.g., “protein digestion and absorption” and “protein processing in the endoplasmic reticulum”) were significantly enriched in both MS vs. C and HS vs. C groups. We also analyzed the expression difference of the non-shared annotated genes after stimulation with mild heat stress and high heat stress, 1,494 and 3,032 non-shared annotated genes were identified, respectively (Figure 2A). The KEGG enrichment of the non-shared genes was then visualized by bubble chart based on their FPKM values (Figure 2B). Interestingly, lots of genes were enriched in “IL-17 signaling pathway,” “Osteoclast differentiation,” and “TNF signaling pathway” in MS vs. C group but not in HS vs. C group. And, “Cardiac muscle contraction,” “Apelin signaling pathway,” and “Tight junction” pathway were only enriched in HS vs. C group (Figure 2C).

### Heat Stress Damages the Skin Epidermis and Decreases the Number of Mucous Cells

**FIGURE 3:**
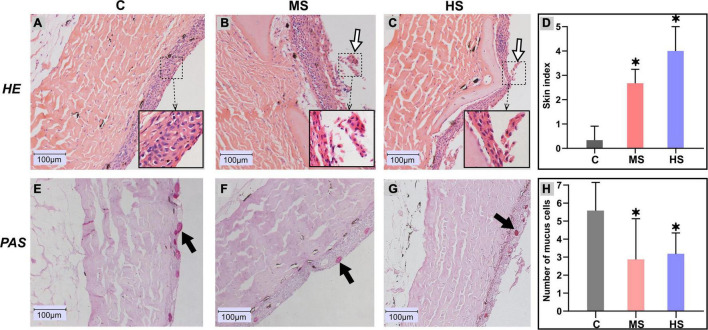
Histological changes in the skin of Siberian sturgeon after exposure to the heat stresses. **(A–C)** indicates hematoxylin and eosin (H&E) staining, **(D–F)** represents Periodic Acid-Schiff (PAS) staining. **(A,E)** 20°C group, **(B,F)** 24°C group, **(C,G)** 28°C group, scale bar = 100 μm. The exfoliated cells are shown by the white arrows, black arrows delegate mucus cells. **(D)** Skin health status (organ index) of sturgeon in different groups. **(H)** Mucus cell count between different groups, *p < 0.05 indicates a significant difference compared to the control group.

To investigate the effect of heat stress on Siberian sturgeon, skin samples were collected for histological observation. The histological observation of the skin samples are shown in Figure 3. The results showed that the epithelial layer of the MS group was exfoliating and necrotizing with a raise of cell debris compared to the control group (Figure 3B). The epidermal layer of the skin in the HS group was partially broken, with slight dissociation and epidermal shriveling (Figure 3C). The pathological scores also indicated significant pathological changes in the MS and HS groups (Figure 3D). PAS staining revealed that the number of mucous cells had decreased significantly in the MS and HS groups compared to the control group (Figures 3E–G). Significant difference in the number of mucous cells from different group also confirmed these results (Figure 3H).

### Characteristics of 16S rRNA Sequencing and the Operational Taxonomic Unit Distribution

A total of 107,557 valid sequences were obtained from the nine samples. In addition, the number of valid sequences in the skin samples ranged from 11,673 to 22,761. The rarefaction curve reached the plateau phase and the rank abundance curve was a long and flat broken line, indicating that the sequencing depth, richness, and evenness met the sequencing requirements (Supplementary Figure 4). The raw 16S rRNA sequencing data have been submitted to the National Center for Biotechnology Information (NCBI) with Accession number PRJNA768965.

**FIGURE 4:**
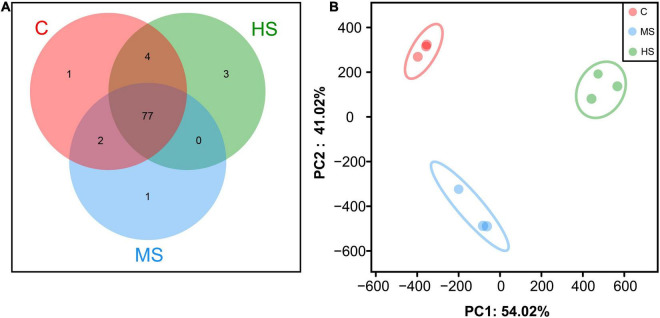
Species composition analysis among different groups. **(A)** Venn comparison diagram of different groups. **(B)** Principal Component Analysis (PCA).

The distribution of OTUs in different groups is shown in Figure 4. Seventy-seven OTUs were shared by sturgeon under the three temperatures, and microbial diversity decreased slightly in group MS compared to the control group, while HS group was similar to the control group (Figure 4A). The β-diversity analysis revealed that all samples were divided into three relatively independent categories, indicating that the diversity of microorganisms varied between the groups (Figure 4B), which was consistent with the Venn diagram results.

### Heat Stress Disrupts the Composition of the Skin Microbiota

**FIGURE 5:**
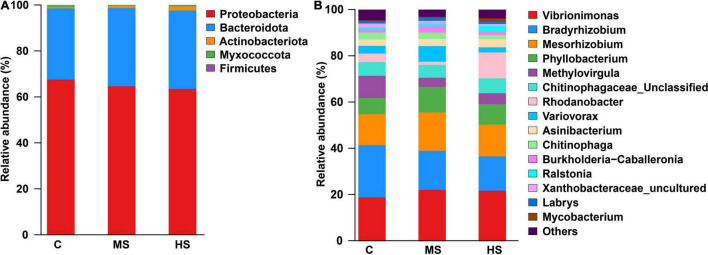
The relative abundance of phylum **(A)** and genus **(B)** under the heat stresses, only classes that are present at abundances >1% in at least one sample are shown.

Analysis of microbial composition showed that the heat stresses induced significant changes in skin microbial composition. The most abundant bacterial types at the phylum level were *Proteobacteria* and *Bacteroidota* under the heat stresses (Figure 5A). At the genus level, we observed a notable decrease in *Methylovirgula* after heat stress, while the *Rhodanobacter* was significantly higher, particularly in group HS after heating up, with a relative abundance of 11% (Figure 5B).

### Heat Stress Reduces the Abundance of Probiotics and Raises the Abundance of Potential Pathogen

**FIGURE 6:**
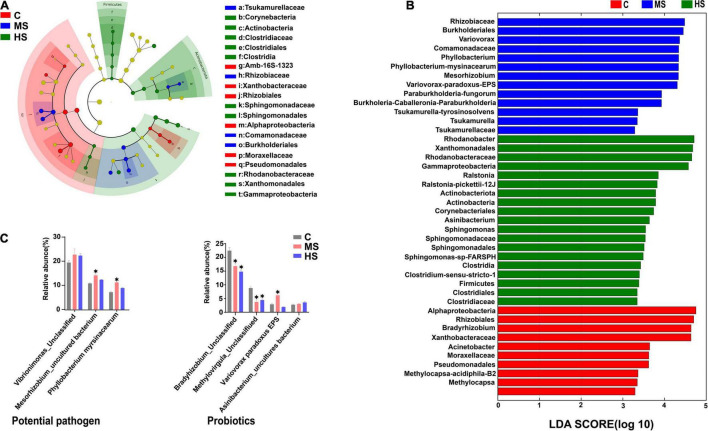
LEfSe analysis of the differences in skin microbiota of Siberian sturgeon. **(A)** Taxonomic characterization of differences in skin microbiota of Siberian sturgeon under different temperature treatments, the concentric circles from the inside to the outside stand for different taxonomic classes (phylum to family). Different color areas point to different groups (the red nodes in the branches represented microbial taxa that play an important role in the control group, the blue nodes indicate microbial taxa that plays a vital role in the MS group, the green nodes mean microbial taxa that play a key role in the HS group, while the yellow nodes show no significant difference). Abundance of skin microbiota were revealed by the size of each node. **(B)** Linear discriminant analysis (LDA) for three groups, scores for remarkable abundant of skin microbiota. **(C)** The relative proportion of species under different temperatures, only classes that are present at abundances >1% in at least one sample are shown. *p < 0.05, indicates a significant difference compared to the group C.

Linear discriminant analysis effect size analysis was carried out to further evaluate the differences in the structure of the skin microbiota in Siberian sturgeon under the heat stresses. The results showed significant differences in the skin microbiota between the heat stress groups and the control group (Figures 6A,B). We screened the microbiota with relative abundances >5% into two major categories of potential pathogen and probiotics according to their functions to further verify this difference at the species level (Figure 6C). Although there were exceptions in the minority of the microbiota, the relative abundance of potential pathogen (e.g., *Vibrionimonas, Mesorhizobium*, and *Phyllobacterium*) tended to increase, while the relative abundance of the dominant probiotic (*Bradyrhizobium* and *Methylovirgula*) decreased significantly.

## Discussion

In this study, transcriptome, histopathological observation and microbiota were analyzed after the heat stresses in the skin of Siberian sturgeon. Transcriptome analysis determined that “keratinocyte differentiation” and “keratinization” terms were highly enriched in the GO enrichment analysis of DEGs of MS vs. C group (Supplementary Figure 3). Differentiation of keratinocytes is a process in which epidermal cells convert into protective stratum corneum, providing skin with the ability to retain moisture (Elias, 2007; Sumitomo et al., 2019). Thus, heat stress may directly affect the formation of stratum corneum, but the mechanism needs more evidences to be uncovered. In addition, keratinocytes produce countless antimicrobial peptides and chemokines that form an effective immune barrier against environmental pathogens (Furue et al., 2020). In HS vs. C group, the term “regulation of chemokine production” was significantly enriched in the GO enrichment analysis of DEGs. Therefore, it is reasonable to conclude that heat stress may also damage the skin immune system by affecting the secretion of chemokines in Siberian sturgeon.

In KEGG enrichment analysis, the enrichment results of the shared DEGs between MS vs. C and HS vs. C groups showed that the heat stresses influenced “ECM-receptor interaction” pathway (Figure 1B). “ECM-receptor interaction” pathway is closely related to the maintenance of organismal health (Hwang et al., 2020; Sainio and Järveläinen, 2020). Thus, the enrichment in “ECM-receptor interaction” pathway suggests that heat stress may damage the balance of skin health via ECM-receptor interaction pathway. Moreover, the pathways related to protein synthesis (e.g., protein digestion and absorption, protein processing in the endoplasmic reticulum) were significantly enriched, in the same line, “Glycine, serine, and threonine metabolic” and “Mucin type O-glycan biosynthesis” pathways were also significantly enriched. Mucins are a large family of high molecular weight proteins, which are composed of amino acids (such as glycine, serine, and threonine) (Feller et al., 1990; Chakraborty et al., 2011; Ramsey et al., 2016; Bansil and Turner, 2018). And, Mucin O-glycosylation is a common covalent modification of serine and threonine residues of glycoproteins (Van den Steen et al., 1998; Bagdonaite et al., 2021). Therefore, our results indicate that heat stress may affect the synthesis of mucins via amino acid metabolic and O-glycan biosynthesis.

In histopathological observation, the results showed that the heat stresses caused the damages and decreased the number of mucous cells in epidermis. Previous studies had shown that the number of mucous cells was decreased when fishes were exposed to stressful conditions, such as catching and transport (Szakolczai, 1997) and raised salinity (Shephard, 1994). Our results reveal that, like other stresses, heat stress also decreases the number of mucous cells in epidermis. Besides, it is well-known that mucous cells directly reflect the decrease of mucus generation (Bols et al., 2001). Thus, these results provide a potential mechanism of the mucin decrease that the mucin generation was decreased by the damages of epidermis and therefore to reduce the number of mucous cells in epidermis.

The outermost layer of fish skin is epidermis, which reflects the degree of stress because of the direct contact with the surrounding environment (Ross et al., 2019). The symbiotic microbiota colonized on epidermis is believed to closely relate to health situation of host (Chen et al., 2018). It has been demonstrated that water with chemical pollutions (e.g., hypoxia, ammonia, and antibiotics) alters the diversity and composition of the microbial community in cultured systems and, thereby, to affect the microbiota of fish skin (Navarrete et al., 2008; Boutin et al., 2013; Pindling et al., 2018). However, the effects of heat stress on fish skin microbiota have been rarely reported. Our study explored the response of skin microbiota to the heat stresses in Siberian sturgeon. The skin microbiota shared identical colony categories at the three heat stress conditions, but their proportions were different, suggesting that the heat stresses altered the abundance of skin microbiota. Specifically, we observed an increase in the abundance of the potential pathogen (*Vibrio*, *Mesorhizobium*, and *Phyllobacterium*) under the heat stresses. *Vibrio* was reported to impair the innate immune system by reducing the mucus protease activity in fish skin (Guardiola et al., 2014; Cámara-Ruiz et al., 2021). And, changes in the abundance of intestinal pathogenic bacteria, *Mesorhizobium* and *Vibrio*, were associated to pathogen infection (Yang et al., 2017). *Phyllobacterium* is believed to negatively affect the colonization of microbial community in skin (Mei et al., 2020). In contrast, significant changes in the abundance of some probiotic bacteria (*Bradyrhizobium*, *Methylovirgula*, *Variovorax paradoxus* and *Asinibacterium*) were also observed under the heat stresses. *Bradyrhizobium* is believed to have antagonistic effects against 10 pathogenic bacteria (Gabriella et al., 2017). *Methanobacterium* and *Asinibacterium* are considered to produce poly-β-hydroxybutyrates that inhibit the growth of pathogens in the host bacterial community (Defoirdt et al., 2007; Halet et al., 2007; Magalhes et al., 2020). And, *Variovorax paradoxus* is associated with biofilm formation (Fredendall et al., 2020). In summary, the abundance changes in the potential pathogens and the probiotics indicate that heat stress derives adverse influences, which associate to break the balance between pathogens and probiotics in bacterial community of Siberian sturgeon.

The mucus is one of the most important nutrient supplies for skin microbiota. Previous study proved that the variance of the proteome of Atlantic salmon (*Salmo salar*) skin mucus induced change of resident bacterial community (Minniti et al., 2019). In this study, the changes of the skin microbial community were happened when the mucus generation was decreased. Thus, instead of the unconnected events, the decrease of mucus generation is closely associated with the changes of microbial community on the skin of Siberian sturgeon under heat stress.

## Conclusion

In this study, the damaged epidermal layer and the reduced number of mucous cells in skin, and the decreased feeding rate and protein synthesis, were observed under heat stresses at 24 and 28°C. These events point that heat stress influences skin- and mucin-constructed barriers against the surrounding environment. Two possible mechanisms are that: the heat stresses reduced the mucin synthesis by influencing the energy supplements via intestine intake, and decreased the mucin generation by damaging the epidermis and therefore to reduce the number of mucous cells in epidermis. The decrease of mucus generation is closely associated with the changes of microbial community on the skin, reflecting in reducing the abundance of probiotics and raising the abundance of potential pathogen.

## Data Availability Statement

The datasets presented in this study can be found in online repositories. The names of the repository/repositories and accession number(s) can be found below: https://www.ncbi.nlm.nih.gov/, PRJNA731649 and https://www.ncbi.nlm.nih.gov/, PRJNA768965.

## Ethics Statement

The animal study was reviewed and approved by the Sichuan Agricultural University Ethics Committee.

## Author Contributions

SY, WX, CT, and JD: conceptualization. SY, CT, CZ, and XH: data curation. JW and XD: formal analysis. SY, JW, and GL: funding acquisition. SY, WX, CT, QC, YL, CL, and QG: investigation. SY, WX, ML, and LF: methodology. JW, JJ, and WL: resources. XD: software. GL: supervision. WX, DL, ZD, and XH: validation. CT: visualization. WX and CT: writing – original draft. SY, WX, and JW: writing – review and editing. All authors contributed to the article and approved the submitted version.

## Conflict of Interest

The authors declare that the research was conducted in the absence of any commercial or financial relationships that could be construed as a potential conflict of interest.

## Publisher’s Note

All claims expressed in this article are solely those of the authors and do not necessarily represent those of their affiliated organizations, or those of the publisher, the editors and the reviewers. Any product that may be evaluated in this article, or claim that may be made by its manufacturer, is not guaranteed or endorsed by the publisher.

## Footnotes

1
http://trinityrnaseq.sourceforge.net/

2
http://www.blast2go.com/b2ghome

3
http://www.genome.jp/kegg/

4
https://github.com/tanghaibao/Goatools

5
http://kobas.cbi.pku.edu.cn/home.do

6
http://drive5.com/uparse/

7
http://rdp.cme.msu.edu/
