# Supplementary material for: Heat Stress Weakens the Skin Barrier Function in Sturgeon by Decreasing Mucus Secretion and Disrupting the Mucosal Microbiota
Source: Front Microbiol. 2022 Apr 26;13:860079. doi: 10.3389/fmicb.2022.860079 (PMC9087187; doi:10.3389/fmicb.2022.860079)
Supplement: Supplementary file 1 [file Data_Sheet_1.ZIP › Supplementary Material.docx]

**
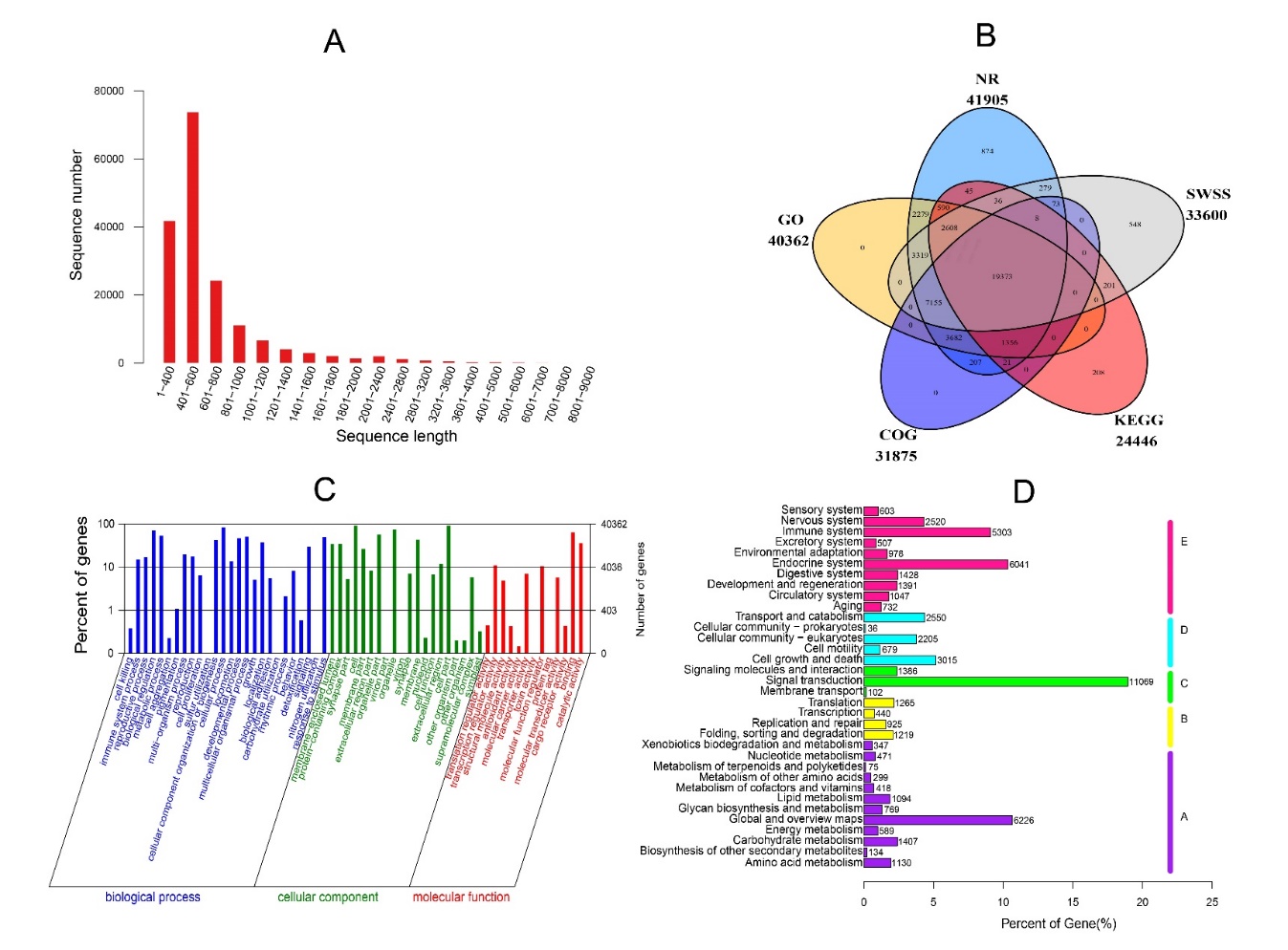
**

**Supplementary Figure 1**

A: The length distribution of Unigenes. B: Statistical graph of gene functional annotation. C: The statistical histogram of GO annotation. The X-axis shows the subtypes of the function annotation, while the Y-axis indicates the number and percent of genes. D: The statistical diagram of KEGG enrichment analysis. The Y-axis presents the enriched KEGG pathways, while the X-axis displays the percentage of the enriched genes among all the genes in pathway. The KEGG pathways are involved 5 types: A, Metabolism; B, Genetic Information Processing; C, Environmental Information Processing; D, Cellular Processes; E, Organismal Systems


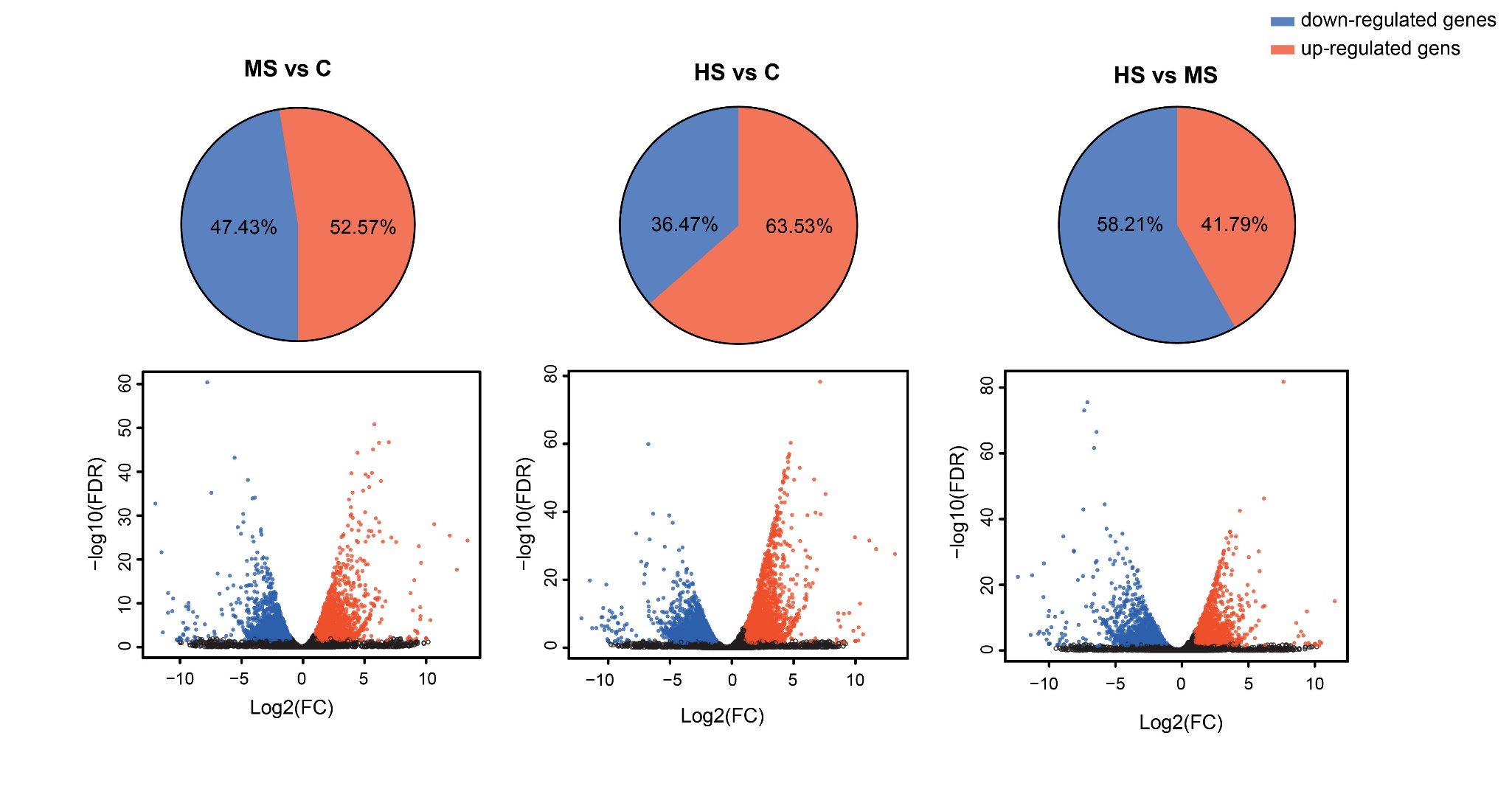


**Supplementary Figure 2**

Volcano plot and statistic of DEGs.
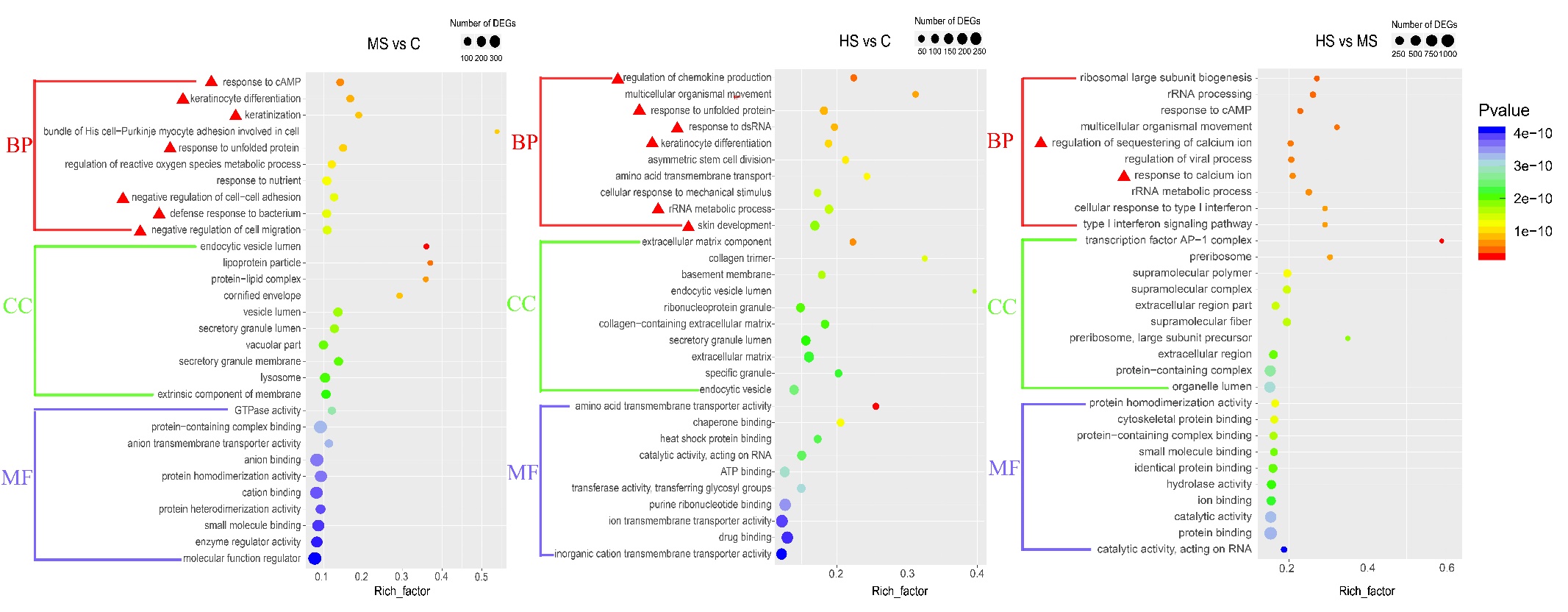


**Supplementary Figure 3**

Scatter diagram of KEGG enrichment analysis of DEGs. The X-axis (rich factor) indicates the enrichment level of DEGs in pathway, while the Y-axis represents the enriched pathways. The size of points shows the enriched number of DEGs. BP: Biological Process; CC: Cellular Component; MF: Molecular Function.


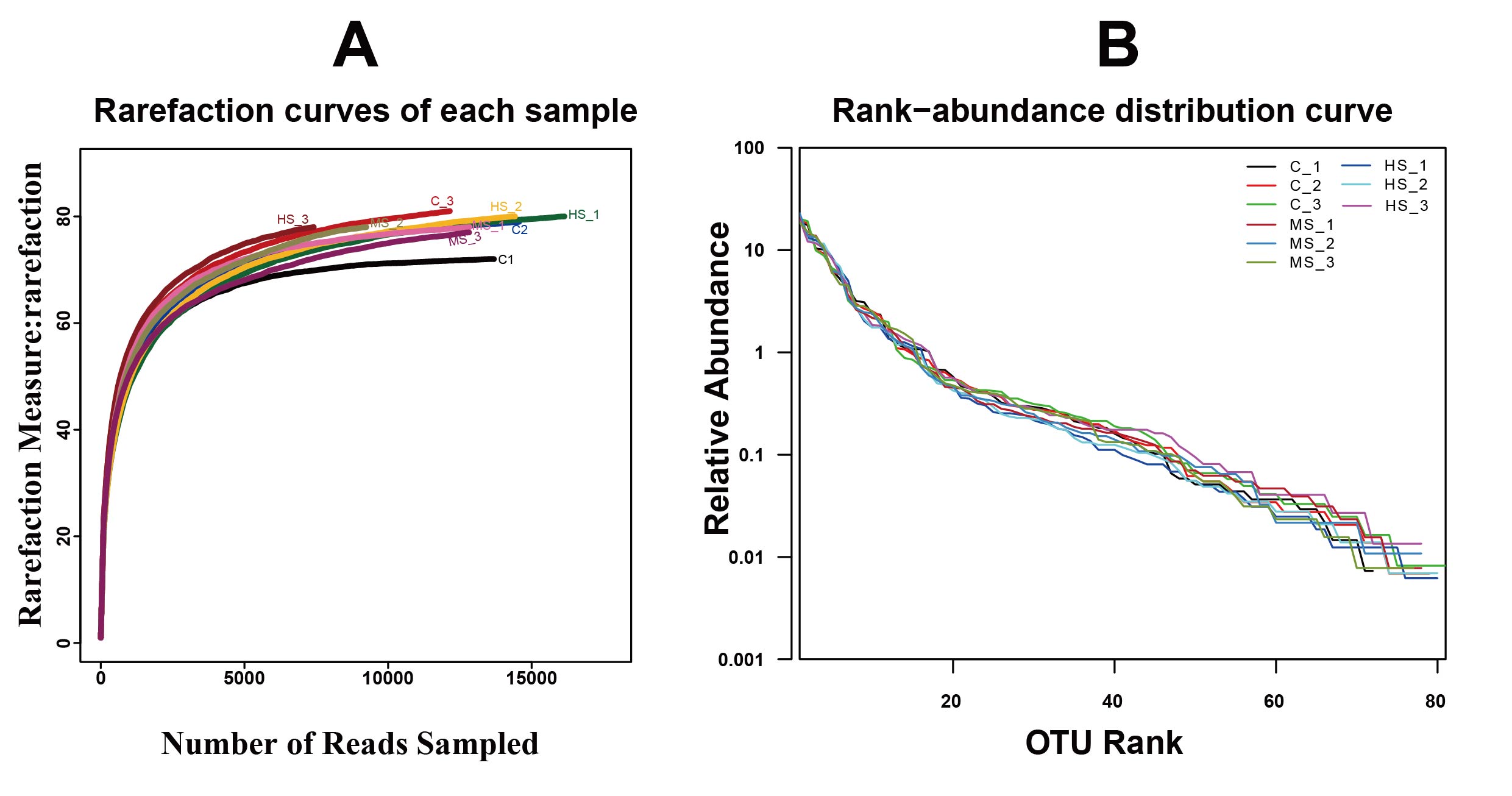


**Supplementary Figure 4**

The rarefaction curves (A) and rank-abundance distribution curve (B) of each sample.
